# Dynamic interactions in the tumor niche: how the cross-talk between CAFs and the tumor microenvironment impacts resistance to therapy

**DOI:** 10.3389/fmolb.2024.1343523

**Published:** 2024-02-22

**Authors:** Oliwia Piwocka, Igor Piotrowski, Wiktoria M. Suchorska, Katarzyna Kulcenty

**Affiliations:** ^1^ Department of Electroradiology, Poznan University of Medical Sciences, Poznan, Poland; ^2^ Doctoral School, Poznan University of Medical Sciences, Poznan, Poland; ^3^ Radiobiology Laboratory, Department of Medical Physics, Greater Poland Cancer Centre, Poznan, Poland

**Keywords:** tumor-microenvironment, cancer-associated fibroblasts, cross-talk, therapy resistance, cancer, radiotherapy

## Abstract

The tumor microenvironment (TME) is a complex ecosystem of cells, signaling molecules, and extracellular matrix components that profoundly influence cancer progression. Among the key players in the TME, cancer-associated fibroblasts (CAFs) have gained increasing attention for their diverse and influential roles. CAFs are activated fibroblasts found abundantly within the TME of various cancer types. CAFs contribute significantly to tumor progression by promoting angiogenesis, remodeling the extracellular matrix, and modulating immune cell infiltration. In order to influence the microenvironment, CAFs engage in cross-talk with immune cells, cancer cells, and other stromal components through paracrine signaling and direct cell-cell interactions. This cross-talk can result in immunosuppression, tumor cell proliferation, and epithelial-mesenchymal transition, contributing to disease progression. Emerging evidence suggests that CAFs play a crucial role in therapy resistance, including resistance to chemotherapy and radiotherapy. CAFs can modulate the tumor response to treatment by secreting factors that promote drug efflux, enhance DNA repair mechanisms, and suppress apoptosis pathways. This paper aims to understand the multifaceted functions of CAFs within the TME, discusses cross-talk between CAFs with other TME cells, and sheds light on the contibution of CAFs to therapy resistance. Targeting CAFs or disrupting their cross-talk with other cells holds promise for overcoming drug resistance and improving the treatment efficacy of various cancer types.

## 1 Introduction

The tumor microenvironment (TME) is a complex and dynamic milieu composed of various cell types, extracellular matrix components (ECM), and signaling molecules that profoundly impact cancer development and progression. TME constituents include cancer-associated fibroblasts (CAFs), immune cells such as tumor-associated macrophages (TAMs), tumor-infiltrating lymphocytes (TILs), endothelial cells, and pericytes. These diverse cell populations interact, influencing many processes crucial for cancer development (Binnewies et al., 2018). CAFs are the predominant cells in the tumor stroma, representing a highly heterogeneous group. Despite much-advanced research, CAFs remain a mysterious TME component that raises universal interest. Normal fibroblasts (NFs) and CAFs are distinct types of fibroblasts playing different roles in an organism. NFs are typically quiscent and maintain tissue homeostasis under normal conditions, while CAFs are activated fibroblasts present in the TME. NFs contribute to tissue repair and maintain its architecture. On the other hand, CAFs support ECM remodeling and create supportive environment for cancer cells (CCs) (Chhabra and Weeraratna, 2023). Most CAFs originate from various cells or become activated from a quiescent state (Piwocka et al., 2023). The best-described pathway of CAF derival is through the interaction of NFs with CCs that secrete cytokines and growth factors that impact fibroblast phenotype. Those factors include tumor-growth factor β (TGFβ), platelet-derived growth factor (PDGFR), fibroblast growth factor 2 (FGF2) (Glabman et al., 2022) or chemokine (C-X-C motif) ligand 1 (CXCL1) (Yamamoto et al., 2023). In normal tissue, TGF-β acts as a cell growth and differentiation regulator, but in the TME, it stimulates cancer progression (Huang et al., 2021). Within tumors, CAFs can originate from several different populations, including epithelial cells through epithelial-to-mesenchymal transition (EMT), endothelial cells via endothelial-to-mesenchymal transition (EndMT), as well as smooth muscle cells, and adipocytes that undergo transdifferentiation. Fibrocytes, originating from monocyte precursors and circulating in the bloodstream, can also transform into CAFs (Liu et al., 2019). Another way for CAFs to arise is through mesenchymal stem cells (MSCs) (Lavie et al., 2022) or cells that preserve progenitor function, such as pericytes, through the PDGF-BB-PDGFRβ loop (Hosaka et al., 2016).There many pathways through which CAFs originate and the cells themselves also present various phenotypes. This heterogeneity poses a significant challenge since there is no unique biomarker for CAFs. Nevertheless, CAFs can be characterized by a set of biomarkers specific to different CAF subsets. Single-cell RNA-sequencing (scRNA-seq) revolutionized our understanding of CAFs and shed light on distinct fibroblast lineages. Recently, scientists divided CAFs based on their function and elevated expression of biomarkers, and proposed the following groups: inflammatory CAFs (iCAFs), myofibroblastic CAFs (myCAFs) or matrix CAFs (mCAFs) (Öhlund et al., 2017), antigen-presenting CAFs (apCAFs), proliferative CAFs (pCAFs), metabolic CAFs (meCAFs) (Elyada et al., 2019; Patel and Singh, 2020; Wang et al., 2021b; Lavie et al., 2022; Ma et al., 2023a), developmental CAFs (dCAFs) and vasculature CAFs (vCAFs) (Bartoschek et al., 2018). In Table 1, we collected biomarkers specific to each subset. While most of these subsets contribute to tumor progression, interestingly, there is also a tumor-inhibiting subset called cancer-restraining CAFs (rCAFs) that are Meflin positive and supposedly can soften cancer stroma, however, more research is needed in this matter (Fang et al., 2023; Yamamoto et al., 2023; Zhang et al., 2023). Costa et al. divided breast cancer (BC) CAFs into four subsets S1-S4 (Table 1) based on the expression of fibroblast activation protein (FAP), caveolin-1 (CAV-1), CD29, fibroblast specific protein 1 (FSP-1), PDGFRβ, and alpha-smooth muscle actin (αSMA). Only subsets S1 and S4 showed a clear pattern of immunomodulation, hence, further research is needed. Moreover, CAFs-S2 were negative for most of the markers, thus, authors speculate that this might comprise another cell type (Costa et al., 2018).

**TABLE 1:** CAF subsets and their biomarkers.

CAF subset	Function	Associated biomarkers	Pathways	References
iCAF	Complement activation, chemokine production, inflammatory response	APOD^+^, C7^+^, CDX2^+^, αSMA^low^, IL-6^+^,	JAK-STAT, NFkβ, TNFα	Öhlund et al. (2017), Patel and Singh (2020), Luo et al. (2022), Ma et al. (2023a)
mCAF/myCAF	Angiogenesis, wound healing, ECM remodeling, organization, biosynthesis of collagen	COL10A1^+^, POSTN^+^, MMP11^+^, SDC1^+^, αSMA^+^,	PTK2, FAK, PDGF, MET	Öhlund et al. (2017), Ma et al. (2023a)
apCAF	Activate CD4^+^ T cells in an antigen-specific manner, immune-modulatory effects	HLA-DRA^+^, HLA-DRB1^+^, CD74^+^	-	Elyada et al. (2019)
meCAF	Metabolic reprogramming (enrichment in glycolysis, metabolism of alanine, aspartate, and glutamate)	PLA2G2A^+^, CRABP2^+^, LDHB^+^	MYC	Lavie et al. (2022), Luo et al. (2022), Ma et al. (2023a)
pCAF	Regulation of cell cycle, IFN-I production	CENPF^+^, NUSAP1^+^, PTTG1^+^, STYMN1^+^, TOP2A^+^, TUBA1B^+^, MYBL2^+^, E2F2^+^	-	Galbo et al. (2021), Ma et al. (2023a)
vCAF	Vasculature development	Nidogen-2^+^, CD248^+^	-	Bartoschek et al. (2018)
dCAF	Production of basement membrane products and paracrine signaling molecules	SCRG1^+^, CD10^+^, Gpr77^+^, SDE genes^+^	-	Bartoschek et al. (2018)
Breast cancer				
CAF-S1	Attract and retain CD4^+^CD25^+^ T cells, increase CD25^+^FOXP3^+^	CAV-1^low^, CD29^med^, FAP^high^, FSP-1^low-high^, αSMA^high^, PDGFRβ^med-high^	OX40L, PD-L2, JAM2	Costa et al. (2018)
CAF-S2	Unknown	CAV-1^neg^, CD29^low^, FAP^neg^, FSP-1^neg-low^, αSMA^neg-low^, PDGFRβ^neg^	-	Costa et al. (2018)
CAF-S3	Accumulate in juxta-tumors, share features with non-neoplastic cells	CAV-1^neg-low^, CD29^med^, FAP^neg^, FSP-1^med-high^, αSMA^neg-low^, PDGFRβ^med^	-	Costa et al. (2018)
CAF-S4	Opposite effect to S1	CAV-1^neg-low^, CD29^high^, FAP^neg^, FSP-1^low-med^, αSMA^high^, PDGFRβ^low-med^	-	Costa et al. (2018)

The intricate cross-talk between CAFs and therapy resistance involves dynamic interactions within the TME. CAFs communicate with cancer cells through the secretion of signaling molecules, activating pathways that enhance cancer cell survival and resistance to therapeutic interventions. Additionally, CAFs contribute to therapy resistance by remodeling the extracellular matrix, creating a protective niche for cancer cells, and modulating the immune response within the TME, establishing an immunosuppressive environment that hampers the efficacy of immunotherapy (Mao et al., 2021). Furthermore, metabolic interactions between CAFs and cancer cells provide alternative energy sources and promote cell survival under the stress induced by chemotherapy or targeted therapy (Jung and Le, 2021). Understanding these complex interactions is essential for developing targeted therapies that can overcome or prevent these resistance mechanisms, ultimately improving the effectiveness of cancer treatments.

Considering the multifactorial role of CAFs, especially in tumor progression, it is crucial to use an appropriate culture system to study those interactions. Developing three-dimensional (3D) cancer models offers ethical and economic advantages in research predicting tumor response to chemotherapy and radiation, bridging the gap between two-dimensional (2D) and *in vivo* studies. Among these 3D models, multicellular tumor spheroids (MCTS) are the most commonly used *in vitro* for preclinical cancer research (Yakavets et al., 2020). The spheroid cancer model recapitulates conditions inside the tumor, including hypoxia and cell-cell or cell-matrix interactions. Moreover, it preserves cell morphology and physiology compared to cells growing as a monolayer (Ryu et al., 2019).

In this review, we aimed to gather the latest information on interactions between CAFs and other TME cells, mainly including research conducted on 3D models. Our paper provides insight into cross-talk with cancer cells, macrophages, endothelial cells, and adipocytes. Those interactions are crucial for angiogenesis, ECM remodeling, metabolism, and therapy resistance. Interactions between CAFs and other immune cells have been described in several reviews (Binnewies et al., 2018; Mao et al., 2021; Raskov et al., 2021). The authors of these reviews describe in detail the interplay between immune cells and cancer microenvironment, which is why we will focus on the interactions that might impact the response to therapy.

## 2 Cross-talk of CAFs and TME cells

### 2.1 CAFs and cancer cells

Cross-talk between CCs and CAFs is a critical aspect of the TME and plays a significant role in cancer progression and drug resistance. CAFs can influence CCs through several pathways, i.e., paracrine signaling, reciprocal feedback, exosomes (Sarkar et al., 2023), and organelle transfer (Nocquet et al., 2020). Additionally, CAFs are recognized for altering the ECM for tumor advantage, making it more suitable for CCs invasion, where interleukins (ILs) and TGF-β play a fundamental role (Asif et al., 2021). One of the best-researched molecules in cancer progression is interleukin 6 (IL-6), which is crucial for proliferation, inflammation, and metabolism (Manore et al., 2022). Dittmer et al. found that CAF-derived condition medium (CM) is rich in IL-6 and compared its action to recombinant human IL-6 (rhIL-6) in the culture of BC cell lines. Both CM and rhIL-6 induced STAT3 phosphorylation (Dittmer et al., 2020), which is known to affect BC progression and metastasis (Ma et al., 2020). Moreover, while in 2D culture, IL-6 promoted apoptosis, in 3D-spheroids, it exhibits an anti-apoptotic effect (Dittmer et al., 2020). Another IL associated with inflammation and immune response is IL-33, which guides Th2 immune reaction. The co-culture of CAFs and gastric CCs revealed elevated migration and invasion of CCs mediated by CAF-derived IL-33 by activation of the ERK1/2-SP1-ZEB2 pathway. What is more, CAFs can be stimulated to release IL-33 through pro-inflammatory cytokines, particularly TNF-α, which is produced by gastric CCs via the TNFR2-NF-κB-IRF-1 signaling pathway (Zhou et al., 2020).

**FIGURE 1:**
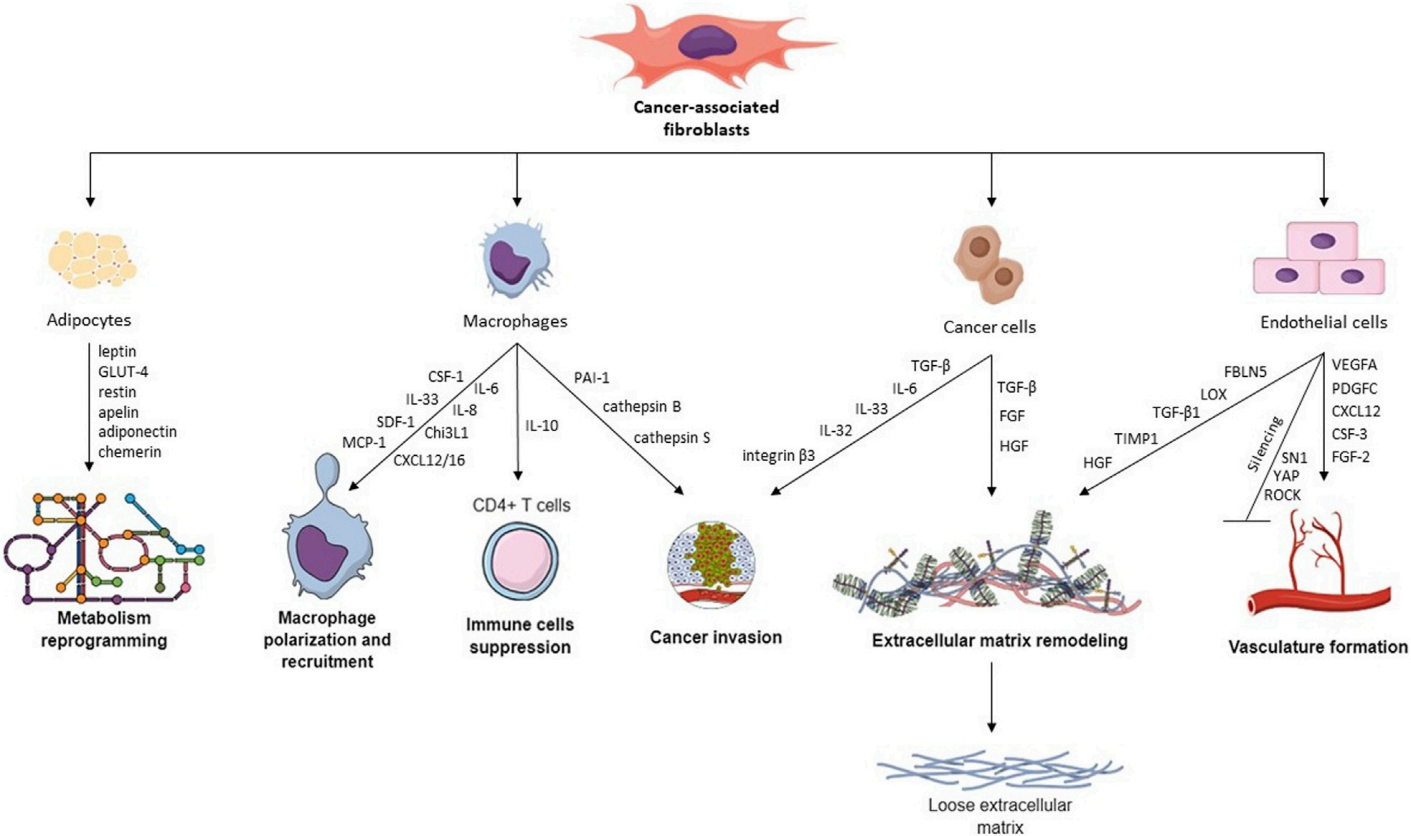
Hallmarks of CAFs and their cross-talk with other TME cells. CAFs interact with different TME cells via various cytokines, proteins, and transcription factors, leading to macrophage recruitment, metabolism reprogramming, cancer invasion, ECM remodeling, vasculature remodeling, and immune cell suppression.

ILs and integrins are not directly connected on the molecular level, however, both can influence cellular and physiological processes, especially cell adhesion, migration, and tissue remodeling. Wen et al. indicated that interaction between CAF-derived IL-32 and integrin β3 facilitates communication of CAFs and BC cells, leading to enhanced invasion (Figure 1) (Wen et al., 2019). Another group researched the long-term (few weeks) effects of CAF-CM on BC cells treated with fulvestrant, an estrogen receptor antagonist. Their research revealed that continuous exposure of these cells to CAF-CM did not enhance their resistance to fulvestrant. In fact, when CAF-CM was absent, the resistance to fulvestrant was decreased. Furthermore, the application of CM increased the expression of integrin β1 in BC cells, and cells developed a dependency on this protein despite it providing no advantages in terms of their ability to tolerate fulvestrant, proliferate, or migrate (Dittmer and Dittmer, 2018). One of the CAFs’ essential abilities is cytokine secretion, including TGF-β, which induces EMT and promotes cell proliferation (Asif et al., 2021). Haga et al. examined the crosstalk between CAFs and CCs in the oral squamous cell carcinoma (OSCC) 3D model, which included cells grown in collagen matrices. They found that CAF-secreted TGF-β upregulated expression of SOX9 in OSCC cells, which supported cancer invasion *in vitro* and *in vivo.* Knockdown of SOX9 in CCs resulted in inhibition of migration and invasion, despite the presence of CAFs, leading to a conclusion that TGF-β invasion depends on SOX9. Moreover, tumors containing CAFs in mice models were larger with irregular margins than tumors with normal fibroblasts (Haga et al., 2021). Another group demonstrated that TGF-β1-activated CAFs enhance tumor invasion, pulmonary metastasis, and EMT by autophagy and the overexpression of FAP-α. FAP-α knockdown reversed EMT and stopped the tumor invasion and pulmonary metastasis induced by TGF-β1-activated CAFs (Huang et al., 2021).

#### 2.1.1 Mitochondrial transfer

One of the core hallmarks of cancer is deregulating cellular metabolism, leading to the uncontrolled growth of tumors. Even when oxygen is available, CCs can change their glucose metabolism, altering energy production primarily towards glycolysis. This metabolic shift results in a state commonly called “aerobic glycolysis” (Hanahan and Weinberg, 2011; Hanahan, 2022). However, CCs often prefer settling in hypoxic conditions, which favors energy production by mitochondria through oxidative phosphorylation (OXPHOS). A growing body of evidence suggests that some solid carcinomas have deregulated mitochondrial metabolism, hence, many studies focus on the mechanism of mitochondrial transfer between cells in TME. Mitochondria transfer usually occurs via tunneling nanotubes (TNTs), extracellular vesicles (EVs), mitochondrial ejection, or cytoplasmic fusion. However, transfer through TNTs is the best described so far. Fibroblasts and mesenchymal cells are the most popular mitochondria donors in cancer, but there are also reported cases among immune cells (macrophages or natural killer T cells) (Sahinbegovic et al., 2020). CAFs can fuel malignant cells by providing intermediate metabolites for mitochondria action. Elevated tricarboxylic acid cycle (TCA) cycle activity by CAFs is associated with malignant cell survival. Additionally, lactate may also stimulate mitochondria formation. Ippolito et al. found that the transfer of CAFs mitochondria appears to selectively influence prostate CCs, particularly those with enhanced invasive potential and a preference for utilizing lactate secreted by CAFs. The study suggests that CAFs’ secreted factors activate a SIRT1/PGC-1α axis in prostate CCs, leading to improved mitochondrial function, increased mitochondrial reactive oxygen species (mtROS) production, and the accumulation of metabolites. Collectively, these events enhance prostate CCs’ migratory and metastatic capabilities (Ippolito et al., 2019). Different groups indicated that CAFs can “teach” BC cells how to promote migration via mitochondrial transfer. To verify the hypothesis, researchers investigated the impact of CAFs on CCs migration by educating BC cells through two methods: co-culture with live CAFs and co-culture with CAFs that later were killed. Early presence of CAFs educated CCs to form spheroids, displaying increased invasive and migratory capabilities. Moreover, ATP levels increased in educated CCs, suggesting altered energy production favoring mitochondrial respiration. CAF mitochondria were artificially introduced into CCs to investigate the role of transferred mitochondria, resulting in increased migration and higher ATP levels. In summary, CAFs educate BC cells via mitochondrial transfer, enhancing their migration and altering energy production (Goliwas et al., 2023). In summary, the available data suggests that mitochondria transfer enables cells to escape apoptosis and acquire therapy resistance.

### 2.2 Macrophages and CAFs

Macrophages are a population of immune cells residing within tissues. They originate from two sources: circulating blood monocytes, which migrate into the tissue where differentiation occurs, and embryonic precursor cells present in the tissue (Das et al., 2015). The role of macrophages includes phagocytosis of microorganisms, modulation of the immune response, and regulation of the functions of neighboring cells. However, their activity heavily depends on their phenotype and the tissue in which they reside (Shapouri-Moghaddam et al., 2018).

Macrophages are also present within tumor tissue (termed tumor-associated macrophages, TAMs), significantly altering this milieu. The available clinical data indicate that the presence of a higher density of TAMs in several cancer types (i.a., gastric, multiple myeloma, breast, ovarian) correlates with significantly worse overall survival (Raskov et al., 2021). However, the macrophages present within TME are not exclusively pro-cancer. Phenotyping methods commonly used in research divide macrophages, including TAMs, into two populations: a pro-inflammatory, tumor-suppressive M1 subtype and an anti-inflammatory, promoting tumor growth M2 subtype (Shapouri-Moghaddam et al., 2018). While M1/M2 division is most commonly used in many studies, other subtypes of macrophages are also described, such as M2a, M2b, M2c, and M2d (Qin et al., 2022). Due to their plasticity, this phenotype is not permanent, and macrophages can change their function depending on external stimuli. What is more, some authors argue that a rigid M1/M2 division of macrophages is overly simplistic and emphasize that the function of macrophages depends on specific activities rather than their perceived M1/M2 state (Ostuni et al., 2015).

Similar to healthy tissue macrophages, TAMs originate from both tissue-resident macrophages and bone marrow-derived monocytes. The origin of TAMs varies depending on the cancer type; for example, in glioblastoma, 85% of the TAM population originate from infiltrating monocytes and 15% from resident microglia (Chen et al., 2017). Data from human tissue, mouse tissue, and spheroid models indicate that in non-small cell lung carcinoma (NSCLC), tissue-resident macrophages are essential for establishing a TME for the early development of cancer lesions (Casanova-Acebes et al., 2021). Similarly, tissue-resident macrophages in BC are crucial for the early development of triple-negative tumors before angiogenesis (Hirano et al., 2023). Furthermore, the macrophages within pre-malignant lesions promote early dissemination of cancer cells and later metastasis, contributing to disease progression (Linde et al., 2018; Hirano et al., 2023). Overall, the available data indicate that tissue-resident macrophages are likely essential in the initial phases of tumor formation, with monocyte-derived macrophages dominating later in tumor development.

The presence of TAMs within TME largely depends on the activity of CAFs. In an *in vitro* study, Ksiazkiewicz et al. (2010) showed that monocyte infiltration was higher in spheres comprising BC cells with primary fibroblasts than in spheres from BC cells alone. This effect is likely caused by factors secreted by CAFs, which differ between cancer types. Some of the CAF-secreted cytokines which are shown to recruit monocytes include CCL2 in lung squamous cell carcinoma (LSCC) (Xiang et al., 2020), IL-33 in lung metastases of BC (Shani et al., 2020), SDF-1 and MCP-1 in prostate cancer (Comito et al., 2013), CXCL12 in prostate cancer (Vickman et al., 2020) and OSCC (Li et al., 2019), MCP-1, SDF-1 (Gok Yavuz et al., 2019) and Chi3L1 in BC (Cohen et al., 2017), CXCL16 in triple-negative BC (Allaoui et al., 2016), IL-8 (but not SDF-1) in colorectal cancer (CRC) (Zhang R. et al., 2019), SDF-1a in hepatocellular carcinoma (Figure 1) (Deng et al., 2017). CCL2 and CXCL12 (also known as SDF-1) are among the most studied and most widely recognized cytokines recruiting monocytes into tumor tissue. Stadler et al. cultured CRC CAFs in organotypic 3D culture and in 2D culture alone, with tumor cells, or with tumor cells and macrophages (Stadler et al., 2021). Interestingly, CCL2, IL-6, and IL-8 production by CAFs were significantly higher when the cells were co-cultured with both macrophages and tumor cells, pointing towards intricate crosstalk required for CAF signaling.

CAF-secreted cytokines not only initiate monocyte recruitment to the tumor but also induce differentiation of macrophages, with most studies indicating a transition towards a pro-tumor M2 subtype. Using a transwell co-culture model, Zhang R. et al. (2019) revealed that CAFs isolated from CRC induce the transition of macrophages toward the M2 phenotype by secreted particles. Similarly, OSCC-isolated CAFs produce factors that induce M2-like phenotype in human monocytes, and importantly, this effect was significantly stronger when CAFs were first stimulated with CM from cancer cells (Cho et al., 2018). CM from cancer cells induced higher secretion of, among others, IL-6 and GM-CSF, which induced differentiation of macrophages, showing that the activity of CAFs depends on CCs.

In a study by Gok Yavuz et al., monocytes cultured with a conditioned medium (CM) from CAF culture induced differentiation towards M2 macrophages, which were capable of suppressing the proliferation of CD4^+^ T-cells and enhanced invasiveness and proliferation of BC cells (Gok Yavuz et al., 2019). Similarly, in hepatocellular carcinoma, CAF-induced M2 macrophages were capable of stimulating cancer cell proliferation, migration, and invasion through secreted PAI-1 (Chen et al., 2021c). In NSCLC, CAFs positive for CD248, a CAF marker correlating with cancer progression, stimulated M2 polarization of macrophages, and in turn, those macrophages induced NSCLC progression (Wu et al., 2022). The role of CD248 in CAFs was also confirmed in patient tissues, where its expression correlated with a worse prognosis. *In vitro* experiments on CRC and pancreatic ductal adenocarcinoma (PDAC) show that CAF and TAM interaction causes the secretion of factors that increase migration, invasion, and proliferation of cancer cells, suggesting induction of a more aggressive phenotype (Zhang et al., 2017a; Stadler et al., 2021).

Interestingly, while CAFs significantly impact macrophages within TME, this interaction is also reciprocated. In prostate cancer, macrophages can induce an activated state in fibroblasts through mesenchymal-mesenchymal transition, converting them into CAFs, which is induced by M2 macrophages more so than the M1 type (Comito et al., 2013). Similarly, in TNBC, monocytes differentiate into immunosuppressive myeloid cells, which activate CAFs (Allaoui et al., 2016). In a study by Zhang et al., *in vitro* co-culture of a macrophage cell line with umbilical cord MSCs resulted in the induction of pro-inflammatory CAF phenotype in these cells (Zhang Q. et al., 2019).

Novel research suggests that macrophages and CAFs are interconnected not only through mutual communication. Tang et al. discovered that macrophages could undergo a macrophage-myofibroblast transition (MMT) and further differentiate into the pro-tumoral subset of CAFs within NSCLC. This process depends on the Smad3 pathway, and the authors showed that targeted therapy through Smad3-blocking could block the MMT and formation of this population of CAFs (Tang et al., 2022).

CAF heterogeneity may impact TAMs within tumors, with different CAF subtypes interacting with monocytes and macrophages distinctly. Several studies point towards iCAFs as the population contributing significantly to creating an immunosuppressive niche, also through their interaction with TAMs. In TNBC patients resistant to immune checkpoint blockade, the population of FAP+ iCAFs secretes CXCL12, recruiting CXCR4+ monocytes into TME (Timperi et al., 2022). In contrast to those results, one of the recent studies on CAF subtypes in gastric cancer showed that extracellular matrix CAFs (eCAFs), not iCAFs, correlate with M2 TAMs (Li et al., 2022). Nevertheless, iCAFs secreted CXCL12 and IL-6, which are responsible for monocyte recruitment. The murine melanoma model showed that S1 iCAFs (Pdpn^high^ Pdgfrα^high^ CD34^high^) produced CXCL12, CSF-1, and CCL8, and also complement components C3 which recruit C3aR+ monocytes (Davidson et al., 2020). Macrophages infiltrating these tumors had higher expression of immune suppressive PDL1, and importantly, targeting the C3a-C3aR pathway resulted in higher infiltration of CD8^+^ T cells, indicating a potential therapeutic target.

Macrophages stimulated by CAFs within the TME affect other populations of immune cells to create the immunosuppressive milieu. One of the roles of inflammatory macrophages is the presentation of antigens for CD8^+^ cytotoxic T lymphocytes (CTLs), which allows for the killing of infected cells as well as tumor cells. TAMs are less capable of effective antigen presentation than inflammatory macrophages, likely due to the lack of crucial co-stimulatory signals (Blohm et al., 2002). While CTLs activated by tumor macrophages lack cytotoxic activity, studies show that TAMs are, in fact, capable of antigen cross-presentation. A recent study by Modak et al. showed that CD206+ M2a macrophages can cross-present tumor antigens to a higher degree than M1 macrophages (Modak et al., 2022). In a recent study, Ma et al. reported the mechanism connecting CD8^+^ T cell immunity with altered methylation pathways in TAMs (Ma et al., 2023b). YTHFD2, an RNA N^6^-methyladenosine (m^6^A) reader, is upregulated in tumor-infiltrating myeloid cells and is likely connected with tumor progression. Knockdown of YTHFD2 in macrophages shifted their phenotype towards more antitumoral and increased TAMs’ ability to present antigens, increasing CD8^+^ T cell immunity.

### 2.3 CAF-derived exosomes

Exosomes are small membrane vesicles that are released by cells into the extracellular environment and play a role in cell-to-cell communication due to their ability to carry nucleic acids, proteins, metabolites, and information via microRNA (miRNA) or non-coding RNA (ncRNA) (Guglas et al., 2022). Exosomes can be released by every cell type and are present in all body fluids (Yang et al., 2019). Exosomes released by CCs often carry specific miRNAs that can be transferred to CAFs, influencing their function and behavior (Suchorska and Lach, 2016). For example, miR-146a transported in exosomes induces differentiation of mesenchymal stem cells to CAFs (Yang Y. et al., 2020). Moreover, miRNAs can modulate gene expression in CAFs, promoting a pro-tumorigenic phenotype characterized by increased secretion of growth factors, ECM remodeling, and enhanced migratory properties. The crosstalk between CCs and CAFs via exosomal miRNAs can contribute to immune evasion and angiogenesis within the TME (Wróblewska et al., 2021; Wyciszkiewicz et al., 2021). CAF-derived exosomes often mediate tumorigenesis since they carry, i.a., TGF-β activating SMAD pathway, CD81 participating in the Wnt pathway, or CD9 participating in MMP2 signaling, leading to ECM remodeling (Peng et al., 2022). Feng et al. delved into the therapeutic potential of CAF-derived exosomal miRNAs, such as miR-29b-1-5p, in gastric cancer (GC) (Table 2) (Wu et al., 2023). His study revealed that miR-29b-1-5p, enriched in CAF-derived exosomes, contributes to GC progression via the VSIG1/ZO-1 axis. Another group found that downregulation of miR-214 led to migration and invasion through FGF9 (Wang et al., 2019a).

**TABLE 2:** Exosomal microRNAs involved in cancer proliferation, progression, and metastasis.

miRNA	Expression level	Function	References
Breast cancer			
miR-92	Upregulated	Promotes immune suppression, proliferation, and migration	Dou et al. (2020)
miR-200s	Downregulated	Induces ECM remodeling by dysregulation of fibronectin (FN) and lysyl oxidase (LOX)	Tang et al. (2016)
miR-500a-5p	Upregulated	Stimulates the growth and migration of BC cells by targeting USP28	Chen et al. (2021a)
Gastric cancer			
miR-29b-1-5p	Downregulated	GC progression via the VSIG1/ZO-1 axis	Wu et al. (2023)
miR-214	Downregulated	Promotes migration and invasion through FGF9	Wang et al. (2019b)
Oral squamous cell carcinoma			
miR-146b-5p	Upregulated	Promotes OSCC malignancy by targeting HIPK3	He et al. (2023)
miR-382-5p	Upregulated	Mediates invasion via RERG/Ras/ERK signaling axis	Sun et al. (2019)
Colon cancer			
miR-200b-3p	Upregulated	Mediates cancer progression via ZEB1, E2F3, IGF1R/PI3K/AKT axis during hypoxia	Gong et al. (2023)
miR-345-5p	Upregulated	Promotes CRC cell proliferation and invasion by targeting CDKN1A	Shi et al. (2023a)
Lung cancer			
miR-196a	Upregulated	Promotes cancer progression via ANXA1 and CCL2	Lee et al. (2021)
miR-210	Upregulated	Overexpresses UPF1 to activate PTEN/PI3K/AKT pathway	Yang et al. (2020a)

Yang et al. focused on BC and the role of exosomal circRNA from CAFs in promoting cancer progression. The study identifies circTBPL1 as a key player in promoting breast cancer cell proliferation, migration, and invasion. The circTBPL1/miR-653-5p/TPBG axis is highlighted as a potential therapeutic target (Ye et al., 2023a). Additionally, exosomal miR-92 originating from CAFs can decrease the expression of large tumor suppressor kinase 2 (LATS2), a crucial element in the Hippo signaling pathway. This results in the elevated nuclear translocation of Yes-associated protein 1 (YAP1), ultimately promoting the transcription of PD-L1 and hindering the proliferation of T cells in BC (Dou et al., 2020). Chen et al. treated BC cell lines with CAF-derived exosomes and found overexpression of miR-500a-5p, which promoted proliferation and metastasis due to attaching to USP28 (Chen et al., 2021a).

He et al. and Xia et al. uncovered the intricate molecular mechanisms behind CAFs-Exo in OSCC (Wang et al., 2023b; He et al., 2023). Studies by He et al. indicated that CAF-derived exosomes were found to modulate OSCC proliferation and immune regulation through specific miRNAs, indicating potential targets for OSCC treatment. Xia’s study delves into the regulatory role of exosomal miR-146b-5p from CAFs in OSCC progression. CAF-derived exosomes containing miR-146b-5p are identified as promoters of OSCC malignancy by targeting HIPK3. Sun et al. co-cultured OSCC cells with CAFs or NFs with or without exosomes. They found increased levels of miR-382-5p, which mediated invasion via RERG/Ras/ERK signaling (Sun et al., 2019).

The investigations of Jing et al. focused on the interplay between microRNA-21-5p (miR-21), hypoxia, and CAFs in head and neck squamous cell carcinoma (HNSCC) metastasis. The study uncovered a signaling pathway involving HIF1α, exosomal miR-21, and CAF activation, suggesting a potential therapeutic target to impede HNSCC invasion and metastasis (Ye et al., 2023b). The role of hypoxia on cancer progression through CAF-derived exosomes has also been confirmed in CRC. Tu et al. investigated the impact of hypoxia on CRC progression through CAF-derived exosomes, specifically focusing on miR-200b-3p. The study uncovered a mechanism involving miR-200b-3p, ZEB1, E2F3, and the IGF1R/PI3K/AKT axis, suggesting a potential therapeutic approach for CRC (Gong et al., 2023). Another group investigating CRC highlighted the influence of CAF-derived exosomal miR-345-5p on CRC progression. The study revealed that miR-345-5p promotes CRC cell proliferation and invasion by targeting CDKN1A, offering insights into potential therapeutic interventions (Shi W. et al., 2023).

In the case of lung cancer, Yang et al. extracted CAF-derived exosomes and proved their contribution to EMT, especially exosomal miR-210, which activated the PTEN/PI3K/AKT pathway associated with migration (Yang et al., 2020a). Fan et al. also investigated the role of miR-210 and revealed that exosome-bound miR-210 secreted from lung cancer cells induces a pro-angiogenic switch in CAFs (Fan et al., 2020). Another group performed profiling of mouse lung fibroblasts and CAFs and selected miR-196a for further study. MiR-196a promoted cancer progression via ANXA1 and CCL2, however, blocking CCL2 restricted invasion within spheroids (Lee et al., 2021).

CAF-derived miRNAs carried by exosomes highly contribute to therapy resistance, which we discussed in Section 3. Furthermore, exosomal carriage properties can pave the way towards precision medicine. Novel research demonstrates that exosomes can be loaded with therapeutic drugs or small molecules targeting specific proteins (Li et al., 2021).

### 2.4 TME in ECM remodeling and vasculature formation

Endothelial cells (ECs) form the inner bedding of blood vessels, playing crucial roles in regulating vessel diameter, cellular movement, and the transport of signaling molecules. Although these cells perform similar functions, their characteristics are often specific to distinct organs (Aird, 2007). An emerging function of CAFs is the ability to initiate vasculature formation dependent and independent through vascular endothelial growth factor (VEGF). CAFs generate factors that stimulate angiogenesis, including VEGFA, PDGFC, FGF2, CXCL12, osteopontin, and CSF3 (Figure 1). These factors aid in developing blood vessels in the TME by attracting myeloid cells, vascular ECs, and monocytes, thereby speeding up the process of tumor angiogenesis. Studies regarding vasculature formation in cell culture require an appropriate angiogenesis model comprising primary mammary ECs, epithelial cells (i.e., MCF-7 cells), and normal or malignant fibroblasts. Experiments on such a model reveal that CAFs increase microvasculature development by generating a malignant microenvironment, compared to NFs (Koch et al., 2020).

Angiogenesis and vascularization would not be possible without ECM remodeling. Sewell-Loftin et al. showed that VEGF is not exclusively responsible for increased vascularization by CAFs. The crucial aspect is the composition of ECM and the concentration of its components, i.e., fibrin, which promotes vessel development. Moreover, pathways such as ROCK, YAP, and SN1 mediate the transduction of mechanical forces needed for remodeling. Silencing of those pathways leads to suppressed ECM remodeling and vascularization (Sewell-Loftin et al., 2017). Herrera and others derived a matrix from PDGF-stimulated CAFs and, on top of it, seeded ECs. ECs formed well-defined capillary networks and tubes in cells, compared to the control group (ECs seeded on matrix from non-stimulated CAFs). Consequently, ECs had upregulated angiogenesis-related genes (phosphor-ERK1/2 and MMP-9) and β1/β3 integrins (Herrera et al., 2018).

ECs can alter their transcriptome during cross-talk with different stromal cell populations, which was visible in phenotypic differences of microvasculature (Curtis et al., 2022). The immune cells of TME are a significant factor in determining the tumor’s vasculature. TAMs promote tumor vascularity through several mechanisms. Degradation of the basement membrane is the first step in vasculature formation, and TAMs secrete matrix metallopeptidase-9 (MMP-9) (Giraudo et al., 2004), which degrades the basement membrane, preparing space for the vasculature (Kessenbrock et al., 2010). More importantly, MMP-9 contributes to an angiogenic switch by increasing the bioavailability of VEGF, which is a crucial factor in angiogenesis (Bergers et al., 2000). Apart from TAMs, other immune cells within the tumor, such as tumor-associated neutrophils (Deryugina et al., 2014) and mast cells (Souza Freitas et al., 2011), are likewise a source of MMP-9. Tumor macrophages are also direct producers of VEGF, along with other angiogenic factors like tumor necrotic factor α (TNFα), basic fibroblast growth factor (bFGF), IL-8, and IL-1 (Dirkx et al., 2006). The formation of tumor microvessels is induced by macrophage expression of thymidine phosphorylase (Kawahara et al., 2010). In gastric cancer, Kawahara et al. demonstrated a correlation between thymidine phosphorylase and microvascular density, however, it did not correlate with overall survival. The immune cells’ impact on vascularity directly translates into the response to therapy. Hughes et al. observed a population of M2 TAMs around tumor blood vessels, which promoted vascularization and subsequent relapse following chemotherapy (Hughes et al., 2015). The VEGF-dependent angiogenesis induced by TAMs can be targeted by VEGF and VEGF receptor inhibitors such as bevacizumab, sorafenib, and sunitinib (Ellis and Hicklin, 2008).

#### 2.4.1 ECM stiffness influence cancer progression

The concept of matrix stiffness in cancer refers to the physical rigidity or stiffness of the ECM. Alterations in the stiffness of the ECM have been observed to influence various aspects of tumor progression and behavior. Solid tumors, such as breast or pancreatic cancer, possess exceptionally stiff ECM. Transcription factors play a pivotal role in these activities, as they react to the stiffness of the matrix and are essential for cellular responses. One extensively researched transcription factor influenced by matrix stiffness is the YAP/TAZ. YAP/TAZ activation occurs in response to a rigid matrix, promoting malignant characteristics in cancer and stromal cells, including CAFs (Ishihara and Haga, 2022). The physiological and chemical remodeling of the ECM during cancer progression involves proteases breaking down the ECM and increased collagen fibril cross-linking mediated by lysyl oxidase (LOX) (Madsen et al., 2015). This raises interstitial fluid pressure, activating CAFs to upregulate TGF-β1 and MMPs, facilitating cancer cell invasion.

Stiffness is a pivotal factor in cancer progression, requiring mechanotransduction for CAF generation and maintenance. The increase in stiffness alone has the potential to stimulate tumor growth. Recent research emphasized stiff TME’s impact on cellular communication network factor 1 (CCN1) regulation in ECs. This regulation elevates the interaction between melanoma cells and endothelium, thereby promoting metastasis through the vasculature (Reid et al., 2017). Pape et al. constructed a 3D model recreating the matrix density occurring in tumors and compared the impact of CAF-rich and healthy stroma on the cross-talk between CCs and ECs. They found that CAFs enhance the surface area of CRC invasion into the stromal compartment. These phenomena were linked to the increased expression of hepatocyte growth factor (HGF), metallopeptidase inhibitor 1 (TIMP1), and fibulin-5 (FBLN5). Interestingly, it hindered the formation of *de novo* vasculature (vasculogenesis) but not angiogenesis. The remodeling process occurred by disrupting endothelial networks, accompanied by an upregulation of VEGFA and a downregulation of vascular endothelial cadherin (VE-Cadherin). VEGFA is involved in acquiring mature blood vessels within the cancer (Pape et al., 2020). Tumors often tend to form aberrant and leaky vessels due to unregulated release of growth factors (Li et al., 2017), hence, blockade of vasculogenesis by CAFs may restrain this mechanism. Vessels within the TME frequently struggle to reach the final stages of maturation, leading to the development of leaky vasculature (Anderson and Simon, 2020). CAFs secrete CXCL12, enhancing local blood vessel analogs’ permeability in the TME. Knockdown of fibroblast-specific CXCL12 reduces vessel permeability, suggesting a role in CAF-mediated ECM remodeling. However, autocrine CXCL12 is not the main initiator of CAF contractility, highlighting its distinct functions in promoting a permeable endothelium that is more prone to angiogenesis and tumor cell intravasation (Holter et al., 2022). Even without soluble CXCL12, CAFs can increase vessel permeability in the nearby TME through alternative mechanisms, like ECM remodeling or reprogramming the TME (Shen et al., 2020). Underdeveloped vasculature and rapid growth of the tumor contribute to an insufficient supply of oxygen and nutrients, which causes cellular stress (Costa-Mattioli and Walter, 2020). The Integrated Stress Response (ISR) is a network of pathways crucial for cellular protein homeostasis and overall adaptations to nutrient starvation on the cellular level. ISR is also activated in cancer cells since some driving mutations like PTEN increase protein synthesis, which requires activation of ISR pathways as a response (Nguyen et al., 2018). As part of the ISR, activating transcription factor 4 (ATF4) regulates protein synthesis, antioxidant production, and autophagy. A recent report showed that in melanoma and pancreatic tumors ATF4 is also important for fibroblast activation, and its knockout inhibits CAF-mediated development of tumor vasculature and tumor growth and metastasis (Verginadis et al., 2022).

### 2.5 Adipocytes and metabolism reprogramming

Adipocytes are the main driving force during the transformation of normal tissue to malignant. They provide energy for regulating various processes in TME and participate in endocrine signaling. Since BC develops near adipose tissue, cancer-associated adipocytes (CAAs) are essential during its progression and metabolic reprogramming due to the secretion of adipokines and energy storage (Brock et al., 2021). Predominantly secreted adipokines include leptin, GLUT4, restin, apelin, adiponectin, and chemerin (Figure 1). Leptin has been shown to play a role in various stages of tumor development, including initiation, growth, angiogenesis, and metastasis. It triggers important pathways such as JAK/STAT, PI3K/Akt, and MEK/ERK1/2. Moreover, leptin causes upregulation of GLUT-1, which increases glucose absorption and modulates glycolysis-associated enzymes, i.a., hexokinase, pyruvate kinase M2 (PKM2), and lactate dehydrogenase A (LDHA). On the other hand, adiponectin exhibits anti-tumor effects by inhibiting SREBPs-dependent lipogenesis and decreases lipid absorption from TME by downregulating CD36 and LDLR. There is also a hypothesis that adiponectin activates autophagy by promoting lipid degradation through β-fatty oxidation (Pham and Park, 2021). Picon-Ruiz et al. found that co-culture of adipocytes with BC cells leads to the release of IL-8, IL-6, IFNγ-inducible protein 10, CCL2, and CCL5. Once immature adipocytes produce those cytokines, it can cause tumor initiation and metastasis (Picon-Ruiz et al., 2016).

Cancers with an adipose-rich TME may exploit fatty acids as the source to stimulate tumor growth and survival (Peng et al., 2021). Adipocytes produce free fatty acids (FFAs), which are then transferred to cancer cells to generate ATP through β-oxidation (Jung and Le, 2021). CAFs can mediate lipid metabolism reprogramming and store more fatty acids and phospholipids to adjust to the deficiency of nutrients in the TME. The lipidomic profiling of CAFs CM revealed increased phospholipids, fatty acids, and cholesteryl ester levels. A deeper analysis of fatty acids in comparison to NFs indicated an increase in diglycerides, phosphatidic acid, phosphatidylinositol, lyso-phophatidylcholines, and phosphatidylethanolamines, demonstrating that CAFs go through lipidomic reprogramming (Gong et al., 2020).

Cancer cells’ metabolism adapts to the microenvironment in which the cells reside. One of the most significant changes is the shift from oxidative phosphorylation towards aerobic glycolysis, even in normoxic conditions. This effect is called the Warburg effect and has been thoroughly researched and described (Vaupel et al., 2019). Since glycolysis is less energy efficient, cancer cells consume much more glucose, a characteristic that can be used for diagnostic purposes. The metabolism of cancer cells is also co-regulated with other cells within TME. While cancer cells can absorb glucose and excrete lactate, in CAFs, lactate absorption, oxidation, and reduced glucose absorption are induced (Koukourakis et al., 2006). The tumor-associated endothelial cells express protein related to higher aerobic metabolism, glucose absorption, and reduced lactate intake. The metabolic collaboration between different TME populations is required for cancer growth. Immune cells also adapt to the TME through changes in metabolism. The increased lactate production by cancer cells can shift the TAMs towards a more M2-like phenotype (Colegio et al., 2014). Macrophages in thyroid cancer present with a metabolism shifted towards aerobic glycolysis through the mTOR pathway (Arts et al., 2016). This pathway influences macrophage glycolysis and promotes epigenetic reprogramming through methylation, with both processes contributing further to the inflammatory functions.

### 2.6 Senescent and aging TME promotes tumorigenesis

The aging of the TME introduces many changes that can significantly influence cancer progression and therapeutic responses. With advancing age, there is a tendency for increased cellular senescence, leading to an inflammatory milieu within the TME (McHugh and Gil 2018). Aging and senescence are closely related concepts and are often used interchangeably, but it is important to address some biological differences. Cellular senescence can contribute to age-related pathologies, and the accumulation of senescence cells is associated with some aspects of aging (Campisi 2013). During senescence, cells undergo irreversible growth arrest, while aging refers to a progressive decline in physiological function over time influenced by genetic and environmental factors (Schmeer et al., 2019). Senescence can be induced by DNA damage and cellular stress to prevent the proliferation of damaged or cancerous cells. Low-dose radiation induces senescence phenotype. Senescence CAFs cultured with BC cells supported their viability and showed protective effects against adriamycin via the PI3/Akt pathway (Tsai et al., 2009). Another way of becoming senescent is through metabolism reprogramming. Senescent fibroblasts may control CAF phenotype by the accumulation of ROS that manifests in elevated α-SMA and vimentin levels regulated by the JAK/STAT3 pathway. The possible mechanism suggests that increased ROS levels intensify the activation of STAT3 phosphorylation by inhibiting its negative regulator, SHP2 phosphatase (Li et al., 2022). In lung cancer, many radiotherapy complications are associated with fibrosis, which also might be mediated by senescent CAFs. Meng et al. assessed the impact of senescent CAF-CM on lung cancer cells and discovered greater radioresistance and a significant increase in expression of SOX2, FOS, MYC, CD44, WNT7A, CXCL1, CXCL6, CXCL8, MMP2, BRAF, and KRAS. Moreover, they found a decrease in RT-induced apoptosis, which may be reversed by STAT3 inhibition, stating its essential contribution to radioresistance (Meng et al., 2021).

Studies show that senescent fibroblasts stimulate tumorigenesis and proliferation by expressing senescence-associated secretory phenotype (SASP) (Coppé et al., 2010). In the aging stroma, the local accumulation of SASP cells may create a specific environment crucial for tumors. Research using samples from young and aged human and mouse lungs has revealed that the age-related buildup of senescent cells with SASP can elevate collagen density and crosslinking (Fane and Weeraratna, 2020). Age-related alterations in the ECM composition and stiffness could impede the penetration of therapeutic agents, affecting their ability to reach CCs. Senescent cells impact the distribution of laminins and collagens, affecting inflammatory phenotypes and promoting tumor proliferation (Owyong et al., 2018). Research on prostate tumors in aged mice suggests the involvement of collagen I and gelatinase in vascular in-growth and tumor progression (Reed 2007). Non-senescent aged fibroblasts derived from healthy human donors exhibit characteristics that promote tumor progression. In a mouse model of skin melanoma, younger animals injected with YUMM1.7 cells developed faster-growing primary tumors, while aged mice displayed increased vessel density and elevated lung micrometastases. Aged fibroblasts from human donors over 55 years old, when introduced into organotypic 3D human skin reconstructions, induced greater invasion but less proliferation in melanoma cell lines compared to fibroblasts from younger donors. Proteomic analysis revealed that the secretion of secreted frizzled-related protein 2 (SFRP2), a canonical WNT antagonist, was significantly higher in aged fibroblasts, contributing to increased melanoma cell invasiveness. Treatment with recombinant SFRP2 in young mice further increased tumor angiogenesis and lung metastasis (Kaur et al., 2016). Senescent CAFs derived from aged individuals demonstrate the production of growth-promoting chemokines, such as CCL-5, contributing to enhanced angiogenesis (Eyman et al., 2009).

## 3 CAFs: orchestrators of resistance in the TME

### 3.1 Drug resistance in 2D and 3D cell cultures

While the research on the effects of drugs on complex TME is progressing, it is important to note that not all models are equally suitable for this topic. The essential step in planning an experiment is to choose an appropriate cellular model for the ongoing research, considering the origin of the cells (established or primary cell lines) and their arrangement (2D, 3D, microfluidic, explant). 2D and 3D cultures exhibit significant differences in immune response, intercellular cross-talk, and drug resistance (Boucherit et al., 2020). Moreover, some drugs act differently under exposure to hypoxia occurring in 3D culture. Some chemotherapeutics, i.e., cisplatin, 5-FU, and doxorubicin, rely on oxygen and show lower effectiveness in 3D culture, and tirapazamine is more competent in hypoxic conditions (Hoarau-Vechot et al., 2018). Breslin et al. examined the impact of the HER-targeting drug, neratinib, on HER-positive BC cell lines (BT474, HCC 1954, and EFM192A) in 2D and 3D cultures. Cells cultivated in 3D showed significantly lower viability after 6 days in culture. However, the neratinib efficacy was substantially decreased since cells in 3D exhibited 78%–90% survival, compared to 60%–65% for respective 2D cultures. Analogous results were observed for the chemotherapeutic drug docetaxel. Interestingly, the type of culture did not impact EMT markers. The reason for elevated drug resistance in 3D cells might be the increased activity of the drug-metabolizing enzyme CYP3A4 in 3D cultures (Breslin and Driscoll, 2016). Another critical factor that must be considered is cell-cell and cell-matrix interactions. Cells cultured in 3D usually show greater stiffness, leading to restricted action of drugs and inhibitors (Feng et al., 2022), as presented by Imamura et al., where more compact spheroids exhibited resistance to paclitaxel and doxorubicin (Imamura et al., 2015). Regarding nanoparticle (NP) usage, cells cultured in 2D and 3D also showed various reactions to NPs concentration or ROS accumulation (Musielak et al., 2023). Considering all the mentioned differences, researchers must carefully choose a cell culture model since promising results obtained in 2D culture may not be replicable in 3D.

### 3.2 Interactions between CAFs and TAMs mediate therapy resistance

Macrophages present within the TME may also influence therapy resistance. Understanding the intricate interplay between macrophages, CAFs, and CCs in the context of drug resistance is crucial for developing strategies to overcome resistance and enhance the efficacy of cancer treatments. Targeting the pro-tumoral functions of TAMs or modulating the immune microenvironment are active research areas in developing novel cancer therapies.

There are several pathways through which CAFs and TAMs interact within tumors, and since these interactions are pro-tumorigenic, many research groups tried to suppress these interactions. TAMs are a vital population responsible for immunosuppression within tumors, a major obstacle for immunotherapy, which presents a new therapeutic target (Zhu et al., 2014). Chi3L1, one of the CAF markers in BC related to TAM recruitment, was targeted as a treatment approach in a mouse model (Cohen et al., 2017). Ablation of Chi3L1 inhibited macrophage recruitment and polarization, increased infiltration by CD8^+^ and CD4^+^ T cells, and also reduced tumor growth. Immunosuppression, crucial in tumor progression and resistance to therapy, might also be induced by CAFs. CM from CAFs induces higher expression of an immunosuppressive marker PD-1 in monocytes *in vitro* (Gok Yavuz et al., 2019).

**FIGURE 2:**
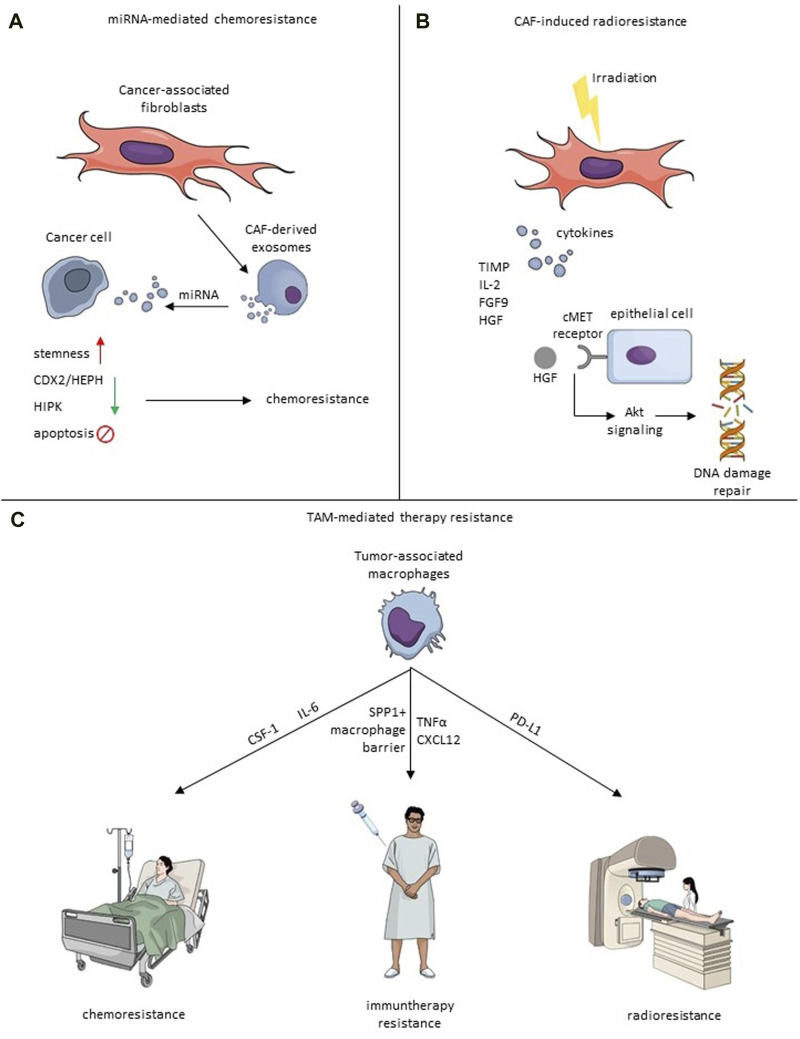
Mechanisms of therapy resistance induced by CAF-TME crosstalk. **(A)** Exosomal miRNA-mediated chemoresistance. Exosomes derived from CAFs secrete miRNA, which impacts cancer cells and causes the increase of stemness, downregulation of CDX2/HEPH and HIPK, or inhibit apoptosis that leads to chemoresistance. **(B)** Irradiated CAFs secrete cytokines, including TIMP, IL-2, FGF9 and HGF. HGF binds to the cMet receptor, which induces an Akt signaling cascade leading to induction of DNA repair. **(C)** TAMs impact chemoresistance via IL-6 or CSF-1, immunotherapy resistance by creating SPP1+ macrophage barrier, CXCL12 and TNFα, and radioresistance through PD-L1.

Targeting the TAMs can be especially appealing not only due to their pro-tumorigenic role but also due to the effect TAMs have on response to therapy. Several pathways in different cancer types have been described as potentially responsible for TAM-mediated chemoresistance, which presents viable targets for new therapeutic approaches. In a xenograft model of BC in mice, TAMs were shown to reduce the effectiveness of chemotherapy (CMF: Cyclophosphamide, Methotrexate, 5-FU) through CSF-1 signaling (Paulus et al., 2006). Crucially, administering anti-CSF-1 significantly reduced the chemoresistance and improved mice survival. TAM-mediated resistance to 5-FU was also observed in CRC patient tissues and mouse models and was mediated by IL-6 secreted by TAMs (Yin et al., 2017). The authors of this study also showed that blocking the IL-6 reversed the protective effect of TAMs. Due to their immunosuppressive character, TAMs can also significantly affect the response to immunotherapy. Targeting PD-1 is one of the most commonly used types of immunotherapy. Research on hepatocellular carcinoma revealed that one of the factors limiting the efficacy of anti-PD-1 treatment might be the structure of the TME (Liu et al., 2023). The authors showed that the tumor immune barrier created by SPP1+ macrophages interacting with CAFs hinders immune infiltration, and targeting the SPP1 improved the efficacy of anti-PD-1 therapy in the murine model of liver cancer (Figure 2). In melanoma, TAM-derived TNFα mediated resistance to MAPK pathway inhibitors, and applying TNFα signaling inhibitor targeted the cancer cells and TME sensitizing the tumor to immunotherapy (Smith et al., 2014). Feig et al. observed that CD8^+^ T cells are present in the murine pancreatic ductal adenocarcinoma (PDA) model. However, administration of anti-α-CTLA-4 and anti-α-PD-L1 therapy alone did not improve the immune killing of cancer cells (Feig et al., 2013). Only when the immune checkpoint therapy was coupled with inhibition of FAP+ CAF-secreted CXCL12 the anti-tumor effect was observed. To evade phagocytosis, tumor cells employ a defense mechanism by upregulating surface proteins known as CD47, which is an anti-phagocytic signal binding to the inhibitory TAM-receptor signal regulatory protein alpha (SIRPα). Notably, CD47 not only prevents phagocytosis but also supports cancer cell proliferation through the PI3K/AKT pathway. The CD47 signaling pathway is recognized as a significant contributor to therapy resistance. Targeting CD47 inhibition emerges as a promising therapeutic strategy, especially when combined with immune checkpoint inhibitors, offering a potential avenue to enhance treatment efficacy (Raskov et al., 2021).

#### 3.2.1 CAF-derived miRNAs in the response to treatment

CAF-derived exosomal (CDE) miRNAs play a crucial role in shaping the TME, specifically focusing on their interactions with CAFs. Understanding the intricate communication network involving exosomal miRNAs and CAFs provides valuable insights into the mechanisms underlying tumor-stromal interactions, offering potential therapeutic targets for disrupting the supportive environment that fuels cancer progression and offering new perspectives for clinical applications in tumor-targeted therapy.

CDEs have been implicated in the response to cancer treatment. Yan’s study focused on the role of CDEs miR-146a-5p in promoting cancer stemness and chemoresistance in urothelial bladder cancer (UBC). Exosomal miR-146a-5p is identified as a potential biomarker for UBC recurrence and a therapeutic target (Table 3) (Zhuang et al., 2023). Another group also examined mechanisms underlying the conversion of stem-cells to cancer-stem cells (CSCs) upon miRNA transfer in BC. They found that CAF-derived exosomes facilitated the transfer of miR-221 to tumor cells. When coupled with hormone therapy, this process activated a feed-forward loop characterized by low estrogen receptor and high Notch expression, contributing to the formation of CD133^high^ CSCs (Sansone et al., 2017). Gao et al. elaborated on how CAFs convey tamoxifen resistance to BC cells. RNA-sequencing provided data about a new CAF subset (CD63^+^ CAFs) that induces tamoxifen resistance. Further investigation revealed that CD63^+^ CAFs secrete miR-22, which causes therapy failure (Gao et al., 2020).

**TABLE 3:** Exosomal microRNAs involved in therapy resistance.

miRNA	Expression level	Function	References
Breast cancer			
miR-221	Upregulated	Hormonal therapy resistance by a generation of CD133^high^ CSCs	Sansone et al. (2017)
miR-22	Upregulated	Induces tamoxifen resistance in CD63^+^ CAFs	Gao et al. (2020)
Colon cancer			
miR-24-3p	Upregulated	Mediates resistance to chemotherapy by downregulation of CDX2/HEPH	Zhang et al. (2021)
miR-770-5p	Upregulated	Induces chemoresistance by downregulation of HIPK1	Zhang et al. (2020a)
miR-590-3p	Upregulated	Elevates radioresistance via CLCA4-dependent PI3K/Akt signaling	Chen et al. (2021c)
Gastrointestinal cancer			
miR-522	-	Inhibits ferroptosis by lipid-ROS accumulation blockage	Zhang et al. (2020b)
miR-27a/b	Upregulated	Induce transformation of NFs to CAFs that mediate resistance to chemotherapy	Tanaka et al. (2015)
Bladder cancer			
miR-146a-5p	Upregulated	Promote cancer stemness and chemoresistance	Zhuang et al. (2023)
miR-148b-3p	Downregulated	Induces chemosensitivity via downregulation of Wnt/β-catenin pathway	Shan et al. (2021)
Lung cancer			
miR-196a-5p	Upregulated	Enhance radioresistance in lung cancer cells by downregulating NFKBIA	Yao et al. (2023)
miR-20a	Upregulated	Restricts PTEN/PI3K-AKT pathway to induce the chemoresistance	Shi et al. (2022)
miR-103a-30	Upregulated	Suppresses apoptosis and promotes cisplatin resistance	Wang et al. (2021a)
Pancreatic cancer			
miR-106b		Promotes gemcitabine resistance of CCs by targeting TP53INP1	Fang et al. (2019)
Ovarian cancer			
miR-296-3p	Upregulated	Activates Akt and STAT3 pathway and promote chemotherapy resistance	Sun et al. (2023)
miR-98-5p	Upregulated	Promotes cisplatin resistance and downregulate CDKN1A	Guo et al. (2019)

Shi et al. investigated the influence of chemoresistant CAF-derived exosomes on CRC progression. The study reveals that exosomes from chemoresistant CAFs promote CRC cell growth and resistance to cisplatin through the delivery of VEGFA (Shi Y. et al., 2023). Methotrexate (MTX) is a chemotherapeutic drug often used to cure CRC, so it is essential to examine its mechanism of action in the treatment of tumors. Zhang et al. found that CAF-derived miR-24-3p mediates resistance to MTX by downregulating the CDX2/HEPH axis (Zhang et al., 2021). Another group revealed the importance of miR-770-5p which induced MTX chemoresistance by downregulation of HIPK1 (Figure 2) (Zhang et al., 2020a).

Yao et al. found that CDEs miR-196a-5p could enhance the radioresistance in lung cancer cells by downregulating NFKBIA, which promoted malignant phenotype. They speculate that this axis may provide a new potential target for lung cancer radiation treatment (Yao et al., 2023). In the case of CRC, radioresistance can be induced by miR-590-3p, which impacts the PI3/Akt pathway (Chen et al., 2021b).

Understanding the intricate interplay between CAFs, their exosomes, and cancer cells has unveiled novel insights into tumor biology and therapeutic opportunities. Collectively, these studies emphasize the potential of targeting CDEs and associated molecular pathways to develop innovative cancer therapies. Future research may further explore the clinical applications of these findings in personalized cancer treatment strategies.

### 3.3 Understanding CAF-mediated radioresistance

Radiotherapy (RT) is undoubtedly one of the most efficient cancer treatments and is currently oriented towards minimalizing damage to non-malignant regions by utilizing, i.a., stereotactic body radiation therapy (SBRT), intraoperative radiotherapy (IORT) or FLASH radiotherapy (Kulcenty et al., 2020; Matuszak et al., 2022; Piotrowski et al., 2022). However, the primary and acquired resistance to radiation exhibited by cancer and stroma cells remains one of the major challenges (Wang Z. et al., 2019). TME plays a pivotal role in tumorigenesis and therapeutic resistance. However, through TME cells, ionizing radiation may even act favorably on tumor progression (Baumann et al., 2016). CAF involvement in resistance to chemotherapy, endocrine therapy, and targeted treatments has been much discussed. Still, recently, CAFs have also been linked to the ineffectiveness of radiotherapy and unfavorable clinical results afterward (Domogauer et al., 2021). Once irradiated, CAFs display unusual radioresistance behavior, as radiation induces cytotoxic effects without resulting in cell lysis (Zhang et al., 2023). Research examining the immediate cytotoxicity of fractionated radiotherapy on CAFs has revealed their intrinsic resistance to radiation. Radiation induces cell-cycle arrest, DNA damage, and p53 activation in CAFs, yet it does not lead to their death (Wang Z. et al., 2019). In BC, irradiated CAFs excrete cytokines protecting them from apoptosis, such as IL-2, fibroblast growth factor 9 (FGF9), brain-derived neurotrophic factor, TIMP metallopeptidase inhibitor 1, and hepatocyte growth factor (HGF) (Wang et al., 2023a). Fibroblasts release HGF, while its receptor, the c-Met receptor tyrosine kinase, is predominantly found in epithelial cancer cells (Jiang et al., 1999), and it mediates AKT signaling cascade activating DNA repair mechanisms and EMT (Figure 2) (Wang et al., 2023a). HGF secretion post-radiation is in line with observations in non-small cell lung cancer (NSCLS) (Berzaghi et al., 2021). Cells not exposed to ionizing radiation can also enhance tumorigenicity with increased levels of HGF. Moreover, BC cells have the ability to modify the behavior of nearby fibroblasts, prompting them to release HGF to promote their development via paracrine signaling (Tyan et al., 2011).

CAFs not only acquire radioresistance but also protect cancer cells from fatal outcomes of radiation. Depending on the applied model, CAFs exhibit various effects regarding CCs survival. The co-culture of CAFs with BC cells caused a delay in tumor development for 3 days after IR, compared to the BC cells alone, which showed a 6-day growth delay, suggesting faster recovery of BC cells once cultured with fibroblasts. Conversely, in the prostate cancer model, such an effect was not observed (Steer et al., 2019). Authophagy plays an essential role in CAF-mediated resistance of cancer cells to radiation. Autophagy is a mechanism that allows a cell to degrade and recycle organelles, proteins, and macromolecules, a process important to cell maintenance and survival in stress (Parzych and Klionsky, 2014). IGF1/2, CXCL12 and β-hydroxybutyrate secreted by CAFs induce autophagy in cancer cells, significantly increasing their recovery after irradiation (Wang et al., 2017). This effect was specific to irradiated cells, and targeting the autophagy pathway mitigated cancer recovery post-irradiation, suggesting a potential therapeutic approach.

CAFs’ multifaceted nature enables them to impact other cells by releasing cytokines and growth factors, but also through metabolism reprogramming. Domogauer et al. found that radioresistance relies on the CAF tissue of origin and is related to the ability to repair DNA single- and double-breaks. The co-culture of CAFs with MDA-MB-231 decreased caveolin-1 (CAV-1) expression associated with oxidative stress, which is one of the hallmarks of cancer. Subsequently, these events increased manganese superoxide dismutase (MnSOD) anti-oxidant activity, contributing to acquired radioresistance (Domogauer et al., 2021). Scientists discovered that CAFs can regulate the expression of superoxide dismutase 1 (SOD1) in esophageal cancer through CXCL1. CXCL1 secreted by CAFs suppressed the expression of the reactive oxygen species (ROS)-scavenging enzyme SOD1, resulting in elevated ROS levels after exposure to radiation. This led to enhanced DNA damage repair and mediation of radioresistance. CXCL1 secreted by CAFs also contributed to radioresistance by activating the Mek/Erk pathway (Zhang et al., 2017b).

IL-6 is a crucial cytokine activating the JAK/STAT3 pathway, participating in BC progression and therapeutic resistance (Manore et al., 2022). Recent research indicated the inherent contribution of CAFs in BC cells in IL-6-mediated radioresistance, which was connected to worse patient survival. However, STAT3 inhibitors and IL-6 antibodies could inhibit this effect, indicating potential in targeting the IL-6/JAK/STAT3 cascade to increase the radiosensitivity of BC cells (Guo et al., 2023).

Radiotherapy, especially delivered at high doses, can increase the immune response against cancer (Finkelstein et al., 2011), however, how radiation impacts the interplay between CAFs and immune cells is not fully understood. Macrophages irradiated *in vitro* with 2 Gy fraction doses present an increased inflammatory phenotype (Teresa Pinto et al., 2016). When CAF-TAM interaction is considered, research shows that CAFs mediated M2-polarization of TAMs after high-dose irradiation, which in turn induced radioresistance in cervical cancer cells (Sheng et al., 2023). Recent research by Gao et al. revealed that irradiation of macrophages also induces immunosuppression by increasing PD-L1 expression (Gao et al., 2023). More importantly, in a mouse model, authors demonstrated that a combination of ATM kinase inhibitor, JAK1/2 inhibitor, and irradiation with 3 doses of 6 Gy resulted in higher CD8^+^ T cell infiltration than irradiation alone. CAFs release several cytokines which suppress the activity of T-cells. It seems that irradiation of CAFs does not strongly modulate this effect–in an *in vitro* study, the secretion of immunosuppressive molecules such as prostaglandin E2, IL-6, IL-10, and TGF-β was not altered after irradiation of lung cancer CAFs (Gorchs et al., 2015). In a similar experiment, the proteins secreted by non-irradiated CAFs and by CAFs irradiated by single high dose or multiple fractions were not different and mediated immunosuppressive effect on macrophages (Berzaghi et al., 2019). The immunosuppressive effect of CAFs on NK cells is also not altered by irradiation, despite the upregulation of inhibitory molecules on the CAF surface (Yang et al., 2021). The abovementioned studies underline that irradiation does not substantially change the composition of CAF-secreted molecules, which is a crucial mechanism of immune suppression. While additional research is necessary, current data show a pivotal role of CAF-TAM crosstalk in tumor progression, and new data suggest that targeting this crosstalk could improve patient outcomes.

## 4 Conclusion

In conclusion, our comprehensive exploration into the intricate crosstalk between CAFs and various cellular components, including cancer cells, macrophages, adipocytes, exosomes, and endothelial cells, within tumor provides valuable insight into the multifaceted dynamics underlying cancer progression, therapy resistance, and metabolic reprogramming. The orchestrated interplay among these diverse cellular entities highlights the complexity of the TME and underscores the significance of considering its holistic nature in understanding and devising therapeutic strategies for cancer. By unraveling the intricate web of interactions, our summary paves the way for more targeted and effective interventions to disrupt key signaling pathways and miRNAs and enhance treatment outcomes in research and therapy.
